# Erector Spinae Plane Block Performed in the Emergency Department for Abdominal Pain: A Case Series

**DOI:** 10.5811/cpcem.1587

**Published:** 2023-08-02

**Authors:** Daniel Parsons-Moss, David Martin, Maribel Condori, Andrea Dreyfuss

**Affiliations:** * Hennepin County Medical Center, Department of Emergency Medicine, Minneapolis, Minnesota; † Alameda Health System - Highland Hospital, Department of Emergency Medicine, Alameda, California; ‡ Hospital Nacional Dos de Mayo, Department of Emergency Medicine, Cercado de Lima, Peru

**Keywords:** Erector spinae block, regional anesthesia, multimodal pain control, ultrasound-guided nerve blocks, case series

## Abstract

**Introduction:**

Ultrasound-guided nerve blocks are fast becoming a core part of opioid-sparing, multimodal, acute pain management in the emergency department (ED) setting. The ultrasound-guided erector spinae plane block (ESPB) has been shown to be effective in treating a variety of musculoskeletal and neuropathic painful conditions in the ED.

**Case Series:**

Here we report the effective use of the ESPB for pain control in four patients who presented with acute abdominal pain related to biliary obstruction in a resource-limited setting.

**Conclusion:**

The ESPB may be helpful in treating abdominal pain related to biliary obstruction, which is a novel indication for this well-established technique. This application is particularly relevant in resource-limited settings with significant delay in definitive surgical management. Further research is needed prior to widespread adoption.

## INTRODUCTION

Ultrasound-guided nerve blocks (UGNB) are a core component of opioid-sparing, multimodal analgesia in the emergency department (ED). The erector spinae plane block (ESPB) is a technique that has been shown to be effective in a variety of painful conditions. The use of this UGNB in the ED was first reported in 2017 by Luftig et al. as an improved alternative to the serratus anterior block for posterior rib fractures.^1^ Since that time, innovative clinicians are finding an expanding list of indications where it may be used successfully to treat visceral pain including pancreatitis, appendicitis, and ureteral colic.^2^–^7^

There is continued uncertainty regarding the exact mechanism of action of the ESPB. The most widely accepted theory is diffusion throughout the fascial plane below the erector spinae muscles and direct action on the ventral and dorsal rami of the spinal nerves.^8^ This provides a reasonable anatomic basis for visceral pain relief as the visceral afferent fibers join the spinal nerves just proximal to the bifurcation of the ventral and dorsal rami, and there is likely some diffusion of anesthetic deep to the erector spinae plane.

Here we present four cases where an ultrasound-guided ESPB was used successfully for visceral pain control in a spectrum of disorders related to biliary obstruction: biliary colic; choledocholithiasis; and gallstone pancreatitis. To our knowledge this is the first report of its use in the ED for pain secondary to biliary obstruction.

## CASE SERIES

### Case 1

A 36-year-old male with no past medical history presented to the ED of a Peruvian hospital for right upper quadrant (RUQ) abdominal pain. He described seven days of intermittent pain that had become constant and more severe for the prior three days. On review of symptoms he also noted dark-colored urine. On physical exam, the patient had jaundice and a positive Murphy’s sign. Laboratory results were notable for elevated white blood cell (WBC) count, normal lipase, elevated bilirubin, and elevated alkaline phosphatase. Point-of-care ultrasound (POCUS) revealed gallstones and a dilated common bile duct, concerning for choledocholithiasis. Subsequent magnetic resonance cholangiopancreatography confirmed the diagnosis of choledocholithiasis.

**Image 1:**
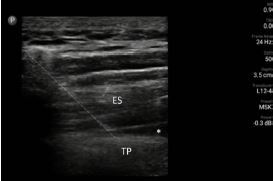
Erector spinae plane block. The dotted line highlights the needle traversing the erector spinae group muscles (labeled ES) in a diagonal trajectory and abutting the posterior side of the transverse process (labeled TP) with a hypoechoic wedge emanating from the tip (asterisk), which is anesthetic spreading along the erector spinae fascial plane.

**Image 2:**
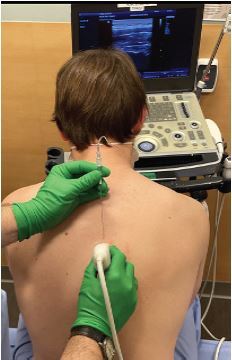
Demonstration of erector spinae plane block technique with the patient in a seated position. Notice the following: 1) ultrasound positioned in front of the operator within direct line of sight; 2) cranial to caudal orientation of the needle (This can be done cranial to caudal or caudal to cranial depending on operator’s comfort and dexterity); and 3) direct in-line placement of the linear high-frequency transducer for good needle visualization.

**Image 3:**
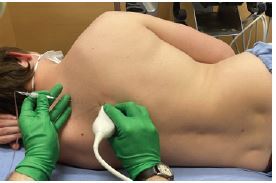
Demonstration of erector spinae plane block technique with patient in a lateral decubitus position.

The patient’s ED course was complicated by severe abdominal pain, subjectively rated 10/10, despite treatment with 100 milligrams (mg) intravenous (IV) tramadol and 1 gram (g) of IV metamizol (a non-steroidal anti-inflammatory drug commonly used in Peru). Given the patient’s refractory pain to opioid and non-opioid IV medications the decision was made to perform an ultrasound-guided ESPB. A high-frequency linear transducer was used to identify the transverse processes at the sixth thoracic (T6) level. Bilateral ultrasound-guided ESPBs were performed using 20-gauge Quincke spinal needles (Becton, Dickinson and Company, Franklin Lakes, NJ) to inject 10 milliliters (mL) of 0.25% bupivacaine and 10 mL of normal saline (NS) using an in-plane approach as shown in Images 1, 2, and 3. Thirty minutes after the procedure the patient’s pain reduced from 10/10 to 0/10 on the self-reported pain scale.

### Case 2

A 25-year-old female, pregnant at 28 weeks estimated gestational age, with a past medical history significant for cholelithiasis, presented to the ED with RUQ abdominal pain. Her pain was described as intermittent and colicky in nature, having been present for one day, and associated with nausea and vomiting. On physical exam, she had tenderness to palpation in the RUQ without rebound or guarding. Lab exams including WBC count, liver enzymes, and lipase were all within the normal reference range. Point-of-care ultrasound was notable for multiple gallstones without signs of cholecystitis or choledocholithiasis. The patient was diagnosed with symptomatic cholelithiasis.

**CPC-EM Capsule**

What do we already know about this clinical entity?

*The erector spinae plane block (ESPB) is an ultrasound-guided regional anesthesia technique useful for treating pain in the emergency department.*

What makes this presentation of disease reportable?

*Here we report the successful use of the ESPB to treat abdominal pain related to biliary obstruction, which is a novel indication for this technique.*

What is the major learning point?

*The ESPB can be used to provide pain control for patients with pain from biliary obstruction in the emergency department.*

How might this improve emergency medicine practice?

*The ESPB is a promising technique that expands the tool kit for multimodal, opioid-sparing analgesia in the emergency department.*

She continued to endorse severe abdominal pain, subjectively rated as 10/10, despite receiving 2 g IV metamizol. An ultrasound-guided, right-sided ESPB was performed using 20 mL of 0.25% bupivacaine and 10 mL of NS. Thirty minutes after the block her pain reduced to 2/10 severity, and she was subsequently discharged home.

### Case 3

A 30-year-old female, with a past medical history pertinent for gallstones and a prior episode of gallstone pancreatitis (10 months prior to presentation), presented to the ED with RUQ abdominal pain and vomiting. Abdominal exam revealed tenderness to palpation in the RUQ and a positive Murphy’s sign. Lab exams revealed elevated lipase and hepatic panel dysfunction with a classic cholestatic pattern. On POCUS, multiple gallstones were visualized without signs of cholecystitis; however, the common bile duct was not visualized. The patient was diagnosed with presumed gallstone pancreatitis.

The patient continued to endorse severe abdominal pain, rated 10/10 severity, despite receiving 100 mg of IV tramadol, and 2 gm of IV metamizol. An ultrasound-guided ESPB on the right side was performed at the T6 level using 20 mL of 0.25% bupivacaine diluted with 10 mL of NS using a similar in-plane approach as previously described. The patient’s pain decreased to a 2/10 severity following the block. She was subsequently admitted for definitive management with endoscopic retrograde cholangiopancreatography followed by laparoscopic cholecystectomy.

### Case 4

A 29-year-old female with no significant past medical history presented to the ED with a two-day history of RUQ pain, associated with nausea and vomiting that started after eating a meal of high fat content. She was noted to be jaundiced, with a positive Murphy’s sign. Lab exams revealed elevated lipase and a cholestatic pattern of liver dysfunction. A POCUS exam demonstrated gallstones. The patient was diagnosed with presumed gallstone pancreatitis.

The patient continued to endorse severe abdominal pain, rated 9/10 severity, despite receiving tramadol 100 mg IV and metamizol 2 g IV. A right-sided, ultrasound-guided ESPB was performed at the T6 level using 20 mL of bupivacaine 0.25% diluted in 10 mL of NS using a standard in-plane approach. Thirty minutes after the procedure the patient’s pain reduced to a level of 2/10. She was subsequently admitted to the hospital for endoscopic retrograde cholangiopancreatography, followed by delayed laparoscopic cholecystectomy.

## DISCUSSION

The ESPB was first described in 2016 by Forero et al. in the anesthesia literature as an effective technique for controlling thoracic pain.^9^ It was first used for rib fractures and bone pain from metastatic breast cancer. Since its initial description, the technique has gained widespread use in postoperative regional anesthesia. Multiple randomized controlled trials have shown it to be effective at reducing postoperative opioid requirements in thoracic, spinal, abdominal (including cholecystectomy), and breast surgeries.^10^ The ESPB technique has since firmly moved into the realm of acute pain management in the ED, where it is finding a growing list of reported indications. However, the literature supporting its use in the ED remains limited. We add this case series to support its use for control of visceral abdominal pain in the ED, which is a new frontier for the technique. When using ESPB for visceral pain there remain several open technical questions: What is the optimal spinal level for injection? What is the optimal volume of local anesthetic? Is bilateral injection superior to right-sided unilateral injection? Further study is needed to answer these questions with certainty.

Control of acute pain is of paramount importance in the practice of emergency medicine. The emergency physician is charged with balancing the goal of patient-centered and syndrome-specific pain control against the risks of pharmacological analgesics, particularly the adverse effects associated with opioid use. Ultrasound-guided nerve blocks have emerged as an important tool for achieving this goal. Additionally, the American Academy of Emergency Medicine and the American College of Emergency Physicians recognize UGNBs as a core component of multimodal analgesia in the ED.^11^,^12^

Performing an UGNB requires a baseline knowledge of POCUS and procedural skills. The cases reported here were performed by emergency physicians during their POCUS fellowship training at a hospital in Peru that has a POCUS fellowship training program, thereby limiting the ability to generalize our findings to other settings with clinicians less experienced in the use of POCUS.

Regional anesthesia techniques such as the ESPB are particularly relevant in resource-limited settings. These patients presented to an urban ED in Lima, Peru, where there is often delay in definitive surgical management due to resource limitations (e.g., case 1), and patients experience extended ED boarding times. However, these conditions are certainly not limited to Peru, and we believe this technique to be very useful wherever it can be safely performed.

## CONCLUSION

In this case series we report successful pain control of visceral abdominal pain related to biliary obstruction with ultrasound-guided erector spinae plane block, a novel indication for this technique in the ED setting. This adds to the mounting body of evidence that ESPB is a useful opioid-sparing technique to control visceral pain in the ED. It is particularly well suited to resource-limited settings, where there may be a significant time delay prior to definitive surgical management of various intra-abdominal conditions. While use of this technique offers great promise to the emergency physician, further research is needed to compare it to other methods of pain control in terms of effectiveness and safety, and to define the optimum technique in terms of vertebral level, right sided vs bilateral, and the quantity of local anesthetic instilled.
